# Supplementary material for: Retrospective cohort analysis of antiretroviral therapy initiation timelines and clinical outcomes in adults with HIV and TB disease in KwaZulu-Natal, South Africa
Source: Int J STD AIDS. 2025 Aug 21;37(1):14–23. doi: 10.1177/09564624251369565 (PMC12783376; doi:10.1177/09564624251369565)
Supplement: Supplemental material - Retrospective cohort analysis of antiretroviral therapy initiation timelines and clinical outcomes in adults with HIV and TB disease in KwaZulu-Natal, South Africa [file sj-pdf-1-std-10.1177_09564624251369565.pdf]

Supplementary Table S1: Univariable and multivariable Poisson regression models of factors associated with successful TB outcomes, among people living with HIV on TB treatment, including patients with unknown TB outcome (N=5,548)

**Time to ART initiation (days)**	**Successful outcome n/N (%)**	**Unadjusted RR (95% CI)**	**Adjusted RR (95% CI)**
≤15	1343 / 1652 (81.3)	1	-
16-56	1577 / 2011 (78.4)	0.96 (0.93-1.00)	0.97 (0.94-1.00)
57-210	387 / 481 (80.5)	0.99 (0.94-1.04)	1.03 (0.97-1.09)
Not initiated within 7 months	630 / 1404 (44.9)	**0.55 (0.52-0.59)**	**0.58 (0.54-0.63)**

Note: Bolded values are statistically significant at $p<0.05$. Successful TB outcome was defined those who completed TB treatment or were cured based on sputum results.

Supplementary Table S2: Univariable and multivariable Poisson regression models of factors associated with successful TB outcomes, among people living with HIV on TB treatment for pulmonary TB (N=4,154)

**Time to ART initiation (days)**	**Successful outcome n/N (%)**	**Unadjusted RR (95% CI)**	**Adjusted RR (95% CI)**
≤15	1233 / 1406 (87.7)	1	-
16-56	1383 / 1618 (85.5)	0.97 (0.95-1.00)	0.98 (0.95-1.01)
57-210	327 / 389 (84.1)	0.96 (0.91-1.01)	1.00 (0.95-1.05)
Not initiated within 7 months	502 / 741 (67.7)	**0.77 (0.73-0.81)**	**0.81 (0.76-0.86)**

Note: Bolded values are statistically significant at $p<0.05$. Successful TB outcome was defined those who completed TB treatment or were cured based on sputum results.

Supplementary Table S3: Breakdown of outcome categories by CD4 count and time to ART initiation (N = 2,816)

Time to ART initiation (days)	Completed n/N (%)	Cured n/N (%)	Failed treatment n/N (%)	Defaulted treatment n/N (%)	Died n/N (%)
**Patients with CD4 <50 cells/µL (n = 575)**					
**≤15**	163 (64.9)	56 (22.3)	1 (0.4)	20 (8.0)	11 (4.4)
**16–56**	197 (64.2)	73 (23.8)	1 (0.3)	21 (6.8)	15 (4.9)
**57–210**	8 (47.1)	6 (35.3)	0 (0.0)	3 (17.6)	0 (0.0)
**Patients with CD4 ≥50 cells/µL (n = 2,241)**					
**≤15**	644 (63.6)	262 (25.9)	4 (0.4)	86 (8.5)	16 (1.6)
**16–56**	737 (63.6)	259 (22.4)	9 (0.8)	123 (10.6)	30 (2.6)
**57–210**	52 (73.2)	5 (7.0)	0 (0.0)	12 (16.9)	2 (2.8)

Supplementary Table S4: Univariable and multivariable Poisson regression models of factors associated with viral load suppression, among people living with HIV who initiated ART with pulmonary TB (N=2,355).

**Time to ART initiation (days)**	**Suppressed n/N (%)**	**Unadjusted RR (95% CI)**	**Adjusted RR (95% CI)**
≤15	780 / 994 (78.5)	1	-
16-56	828 / 1065 (77.7)	0.99 (0.95-1.04)	0.99 (0.95-1.04)
57-210	158 / 215 (73.5)	0.94 (0.86-1.02)	0.93 (0.84-1.02)
Not initiated within 7 months	58 / 81 (71.6)	0.91 (0.79-1.05)	0.90 (0.78-1.05)

Note: Bolded values are statistically significant at $p<0.05$. Successful TB outcome was defined those who completed TB treatment or were cured based on sputum results.
